# Colden’s Liquid Beef Tonic

**Published:** 1885-12

**Authors:** 


### Colden's Liquid Beef Tonic.

We have used this preparation with great satisfaction, and that the medical profession may properly understand its merits, we give the analysis by Dr. Hassell, the distinguished physician and chemist of London, England:

| | |
|---|---|
| 20 per cent. saccharine matter, | 20 |
| 25 per cent. glutinous or nutritious matter obtained in the condensation of the beef, | 25 |
| 25 per cent. spirits rendered non-injurious to the most delicate stomach by the extraction of the fusel oil, | 25 |
| 30 per cent. of an aqueous solution of several herbs and roots, among which are most discernible Peruvian and Calisaya barks, | 30 |
| Total, | 100 |

Dr. Hassell further says:

"I have had the process explained by which the beef in this preparation is preserved and rendered soluble by the brandy employed, and I am satisfied this combination will prove a valuable adjunct to our pharmacopœia."

Since the date of the above analysis, and by the urgent request of several eminent members of the medical profession, two grains of soluble citrate of iron have been added to each wineglassful of this preparation.

